# Efficacy of Green Cerium Oxide Nanoparticles for Potential Therapeutic Applications: Circumstantial Insight on Mechanistic Aspects

**DOI:** 10.3390/nano12122117

**Published:** 2022-06-20

**Authors:** Maarij Khan, Zia-ur-Rehman Mashwani, Muhammad Ikram, Naveed I. Raja, Azza H. Mohamed, Guogang Ren, Ahmad A. Omar

**Affiliations:** 1 Department of Botany, PMAS Arid Agriculture University, Rawalpindi 46300, Pakistan; niazikhan5858@gmail.com (M.K.); drnaveedraja@uaar.edu.pk (N.I.R.); 2 Department of Agricultural Chemistry, College of Agriculture, Mansoura University, Mansoura 35516, Egypt; azza71@mans.edu.eg; 3 Citrus Research and Education Center, University of Florida (IFAS), Lake Alfred, FL 33850, USA; 4 School of Physics, Engineering and Computer Science, University of Hertfordshire, Hatfield AL10 9AB, UK; g.g.ren@herts.ac.uk; 5 Biochemistry Department, Faculty of Agriculture, Zagazig University, Zagazig 44511, Egypt

**Keywords:** cerium metal, cancer, DNA fragmentation, lipid peroxidation, drug delivery, diabetes, antimicrobial

## Abstract

Green synthesized cerium oxide nanoparticles (GS-CeO_2_ NPs) have a unique size, shape, and biofunctional properties and are decorated with potential biocompatible agents to perform various therapeutic actions, such as antimicrobial, anticancer, antidiabetic, and antioxidant effects and drug delivery, by acquiring various mechanistic approaches at the molecular level. In this review article, we provide a detailed overview of some of these critical mechanisms, including DNA fragmentation, disruption of the electron transport chain, degradation of chromosomal assemblage, mitochondrial damage, inhibition of ATP synthase activity, inhibition of enzyme catalytic sites, disorganization, disruption, and lipid peroxidation of the cell membrane, and inhibition of various cellular pathways. This review article also provides up-to-date information about the future applications of GS-CeONPs to make breakthroughs in medical sectors for the advancement and precision of medicine and to effectively inform the disease diagnosis and treatment strategies.

## 1. Introduction

Cerium is an iron-gray lustrous element that belongs to the lanthanide group, with an atomic number of 58 and an atomic weight of 140.166 [1]. Cerium was discovered by the Italian astronomer Giuseppe Piazzi in 1801, and he gave it the name ceres. It is an interesting element due to its electronic structure, which displays two variable oxidation states, Ce^+3^ and Ce^+4^, due to its constantly changing valance electron positions between (d) and (f) orbitals [2]. Naturally, cerium is a solid ductile metal at room temperature. Cerium is an abundant rare-earth element that makes up to 0.0046% of the earth’s crust [3]. Cerium is malleable (hydroscopic) and can be rapidly oxidized at room temperature, especially in the case of high humidity. Cerium is the most reactive earth metal except for europium, which also belongs to the lanthanide category. Cerium decomposes quickly in hot water and sluggishly in cold water [3]. Cerium and its compounds have many applications in different industries. Cerium oxide is used in the walls of self-cleaning ovens, in incandescent lantern mantles, for polishing glass surfaces, and as a decolorizer for glass [4]. Cerium is a good heat and electricity conductor, along with a potential ultraviolet ray absorber [5]. Due to its special electronic structure, cerium varies between cerium dioxide (CeO_2_) and di-cerium trioxide (Ce_2_O_3_). However, Ce_2_O_3_ is an unstable form and is radially converted into CeO_2_ [6].

**Figure 1:**
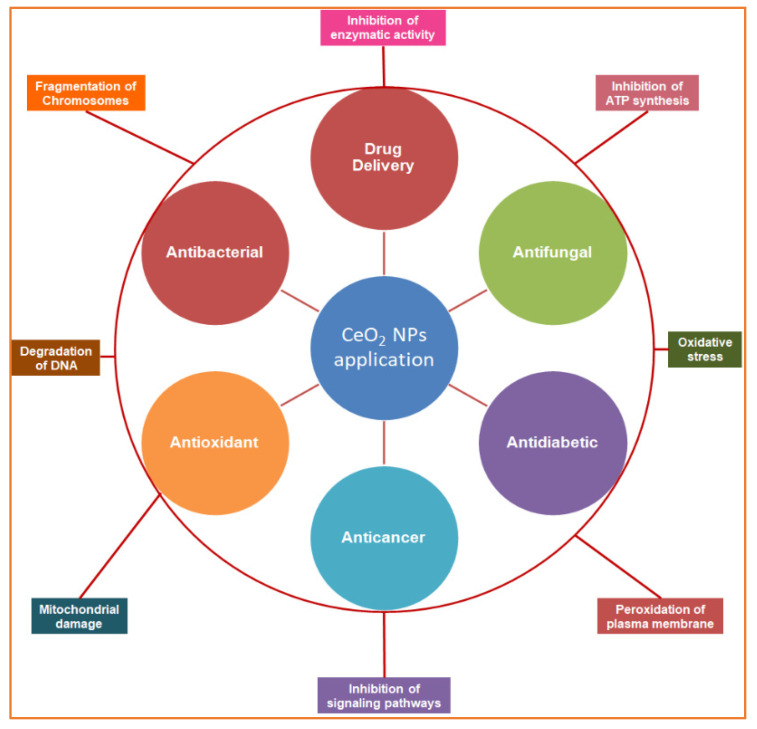
Schematic overview of the study representing the biomedical applications of biogenic cerium oxide nanoparticles (inner circle) and their mechanisms of action (outer circle).

**Figure 2:**
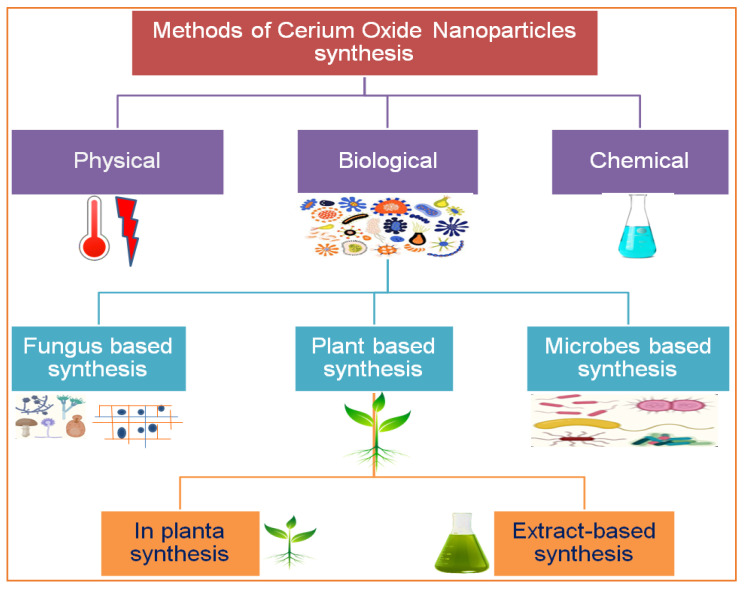
Various routes for the synthesis of cerium nanoparticles.

Nanotechnology is an emerging field of science, and metallic salt was successfully converted into nanoparticles by American Physicist Dr. Richard Feynman, an early pioneer of nanotechnology. An overview of the use of CeO_2_ NPs in the biomedical field and the proposed mechanisms is illustrated in Figure 1. There are numerous methods, tools, and techniques available to synthesize CeO_2_ NPs. Due to their small size and high surface-to-volume ratio, NPs have many applications in different industries, such as pharmaceutical, medical, cosmetic, agricultural, and engineering sectors [7]. CeO_2_ NPs exhibit unique physical and chemical properties that deviate significantly from the bulk metallic salt, and the positive charge on the CeO_2_ NP surface allows it to bind various functional groups [8], which are special because of their biological properties. Cerium is a good oxidizing agent, a substance that gains electrons from others by reducing itself in a chemical reaction. Because of this, researchers found that CeO_2_ NPs can be an important ingredient in the latest medicines that are used against oxidative stress and to treat ailments that arise due to oxidative damage in the human body. Recently, different methods have been implemented to synthesize CeO_2_ NPs, including solution precipitation, spray pyrolysis, sonochemical methods, solvothermal methods, ball milling, thermal decomposition, sol–gel methods, and thermal hydrolysis (Figure 2) [9]. Basically, there are three categories for the synthesis of CeO_2_ NPs: chemical, physical, and biogenic. The chemical and physical methods are both expensive. They require hazardous chemicals, which are not eco-friendly and harmful to the living body, and high temperatures and pressures. The living body vigorously reacts to these chemicals, which can generate major health issues rather than solutions [10]. Our review focuses on the GS of CeO_2_ NPs because plants are factories of useful and essential secondary metabolites. There are 452 plant families, and each plant is a reservoir of primary and secondary metabolites. Plants growing in a xeric environment or at high altitudes always store unique types of phytochemicals. The plant extract is a cocktail of phytochemicals that reduce metallic salt to nanosized particles. [4]. Various organic and inorganic polymers bind through ionic and covalent bonds to NP surfaces. The plant extract is a cocktail of numerous organic polymers [11]. These polymers encapsulate NP surfaces during synthesis and decorate them with various functional groups. The biopolymer coating makes CeO_2_ NPs biocompatible and less toxic entities [12]. Organic coatings stabilize NPs and protect them from nonspecific interactions with cell receptors, proteins, enzymes, and membrane polysaccharides [13]. Plants are small factories of chemicals, and each factory prepares special products that are different from others, such as flavonoids, terpenoids, saponins, glutathione, hydrogen peroxide, ascorbic acid, tannins, caffeine, amines, and nicotine. The coating of these organic compounds on CeO_2_ NP surfaces makes them safe and efficient for medicinal purposes [14]. This review article focuses on the synthesis approaches of CeO_2_ NPs with a special focus on plant-mediated synthetic approaches and various potential therapeutic applications of CeO_2_ NPs. Further, this review article also highlights the various mechanistic routes that CeO_2_ NPs adopt to treat various human diseases.

## 2. Phytosynthesis of CeO_2_ NPs and Other Alternative Approaches

**Figure 3:**
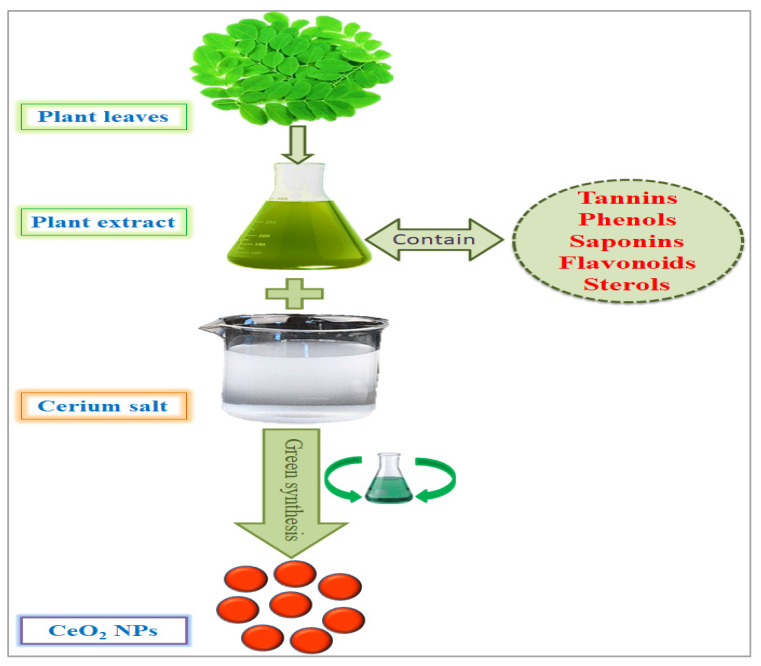
Green synthesis of cerium nanoparticles and encapsulation with plant phytochemicals.

There are several methods used to synthesize NPs. Each synthesis technique follows particular protocols to obtain the specific size and shape of NPs [15]. Some methods require special equipment and temperature and pressure conditions to synthesize NPs. Nanomaterials have been prepared through two standard methods for a long time: physical and chemical (Figure 2) [16]. These two methods are not only health-hazardous but also make it costly to obtain a defined amount of nanomaterial [17]. Recently, researchers developed another method termed ‘green syntheses’ to make nanoparticles (Figure 3). The use of plants to synthesize NPs is beneficial in many aspects; for example, they are cost-effective, non-toxic, easy, safe, and time-saving [18]. The efficacy of medicinal plants for the synthesis of nanoparticles is due to their phytochemical contents, which are involved in the synthesis of various shapes and sizes of nanoparticles. Various phytochemicals present in plants, including flavonoids, polyphenols, alkaloids, terpenoids, and low/high-molecular-weight proteins, are involved in the green synthesis of metallic nanoparticles upon the reduction of their precursor salts into nanoparticles and their stabilization in a redox-mediated process (Figure 3). Each plant stores special phytochemicals. Different plant species contain different phytochemical profiles. Most plants synthesize these chemicals for protection against biotic and abiotic stresses [10]. These plants’ phytochemicals can be ketones, carboxylic acids, phenols, ascorbic acids, amines, carbonyl, hydroxyl, and benzene [19]. The different parts of plants that are used to prepare herbal extracts include roots, stems, leaves, bark, flowers, pollen, or the whole plant body. Many studies are available that have utilized plant-derived biological products, such as resins, gums, nectar, chitosan, and juice, as reducing agents [20]. The use of fresh plant material for extract preparation is superior and health-conscious because the plant body contains many volatile chemicals, hormones, enzymes, vitamins, proteins, and micro and macro trace elements that are not present in plant-derived biological products [21]. During green synthesis, the functional groups present in a plant extract encapsulate NP surfaces and convert them into less toxic, biocompatible, and biodegradable products. One study reported that GS CeO_2_ NPs rarely show drug toxicity and are drug-resistant because of their organic nature [22]. The green synthesis of nanoparticles is considered a good replacement to overcome the challenges of cytotoxicity and genotoxicity [23]. Another study explained that GS CeO_2_ NPs did not produce any allergic reactions in the case of a surgical wound. Three steps are involved in GS CeO_2_ NPs: (i) nucleation/fabrication, (ii) growth/development, and (iii) encapsulation/coating. The first step is the nucleation of metallic salt into same-sized particles; second is the growth and development of nuclei (all nuclei are not formed at once because this causes the agglomeration of NPs), and in the third step, NPs are emulsified and decorated with plant functional groups [24].

In previous research, researchers have used various plants to prepare GS CeO_2_ NPs of different sizes and shapes (Table 1). *Prosopis juliflora* (Sw.) DC. leaf extract-mediated spherical CeO_2_ NPs 15 nm in size were fast enough to cross the cell membrane [25]. Similarly, in another study, *Punica granatum* L. fruit extract was used to fabricate 10 nm spherical CeO_2_ NPs that were small enough to reach the genetic material of cells by crossing the nuclear membrane. *Sida acuta*. whole plant extract-mediated CeO_2_ NPs 8.2 nm in size were found to be a feasible carrier to the blood–brain barrier to deliver drugs to brain cells [25]. The leaf extract of *Justicia adhatoda* L. and *Origanum majorana* L.-mediated CeO_2_ NPs with a 200 nm diameter were observed to be good entities for delivering drugs through the bloodstream [26]. One study demonstrated the use of tannic acid and pectin to synthesize spherical CeO_2_ NPs with 20 nm and 40 nm diameters, which are small enough to deliver drugs inside the cell [27]. Nanomaterials are characterized by different techniques: scanning electron microscopy (SEM) to determine the size of NPs, transmission electron microscopy (TEM) to provide information about shape and surface morphology, UV–visible techniques to measure NP concentrations in a solution, Fourier transform infrared spectroscopy (FTIR) to inform about chemical bonds on the NP surface [28], and energy-dispersive X-ray spectroscopy (EDX) to confirm the chemical nature of the compounds used to synthesize nanomaterials [29].

## 3. Physicochemical Parameters Affecting the Synthesis of Green Cerium Oxide Nanoparticles

Distinct physicochemical reaction parameters, for instance, cerium salt, pH, temperature, and the proportion of biological extract, work collectively to control the molecular dynamics, reaction kinetics, enzymatic reactions, and protein conformations that affect the size, shape, and biochemical properties of nanoparticles [55,56]. Different physicochemical reaction conditions determine the different morphologies of nanoparticles, such as polygonal, cubic, spherical, round, crystalline, and octahedral. Physicochemical parameters perform the function of a toolkit to sculpt and trim the nanoparticles into various sizes and shapes [57]. Biogenic synthesis of nanoparticles is a safe, less toxic technique that has recently been utilized by researchers. This technique uses different biological resources, including plants, microbes, algae, fungi, or any other biologically derived products [58]. These biological extracts are a rich source of biochemicals, such as terpenoids, saponins, flavonoids, amines, ketones, phenols, carboxylic acid, glutathione, hormones, minerals, vitamins, and enzymes that are involved in the reduction of metallic salt into nanomaterial [59]. Plants are considered the agents with the most potential due to their abundance, safe and unharmful nature, and the fact that they are the large factories of phytochemicals/secondary metabolites that are free of chemical danger. Different plant parts, such as leaves, stems, roots, flowers, fruit, pollen, bark, and wood, store phytochemicals of variable nature according to their role in the plant life cycle [58]. For that reason, each plant part contains a variable proportion of phytochemicals that determine the amount of plant material to utilize for the green synthesis of nanomaterials. Secondary metabolites are differentiated into organic and inorganic chemicals, including oil, gum, resins, hormones, nectar, and ascorbic acid (6). This is why the plant body is considered the bank of several functional groups that coat and charge nanoparticles’ surfaces in green synthesis [60]. However standard physicochemical parameter measurements for the green synthesis of nanoparticles vary from plant to plant. Physicochemical reaction conditions participate directly to control the size, shape, and yield of cerium oxide nanoparticles [56]. Temperature is actually the source of energetic electrons, and their flow also energizes other sources that they strike. Temperature provides kinetic energy that accelerates the chemical reaction. Temperature kinetic energy acts as the activation energy that is usually required to start the chemical reaction. Temperature triggers molecular collision that ensures coalescence between the phytochemical extract and cerium salt and converts the solution into the final product [61]. The surface charge of nanoparticles is also controlled by the pH of the solution. The pH value constantly varies during nanoparticle synthesis. Variable pH conditions have a different impact on the reaction kinetics and molecular mechanisms. The increase or decrease in pH value determines the number of H^+^ ions in the reaction solution. A higher pH value is responsible for low H^+^ ions, and lower pH results in more H^+^ available in the reaction mixture. The pH does not contribute to the determination of nanoparticles’ shape and size, but changes in pH influence the electronegative properties and oxidation states by inhibiting the enzyme’s active site, reducing its binding ability, which decreases the rate of the reaction and finally yields nanoparticles [62]. The proportion of reactants directly influences the catalytic property of the reaction mixture as well as the quantity of metallic salt, which both collide in a synergistic way to enhance the nanoparticle yield. The proper mass of the metallic salt and the appropriate volume of the plant extract ensure the presence of an equitable number of reducing and oxidizing agents in the reaction mixture [63]. The selection of physicochemical reaction parameters influences the morphological, physiochemical, and charge-carrying properties of cerium oxide nanoparticles, which affect their biocompatibility, bioaccessibility, biodegradability, and reactivity for treating different diseases [64].

## 4. Green Cerium Oxide Nanoparticles as Strong Antioxidant Agents

**Figure 4:**
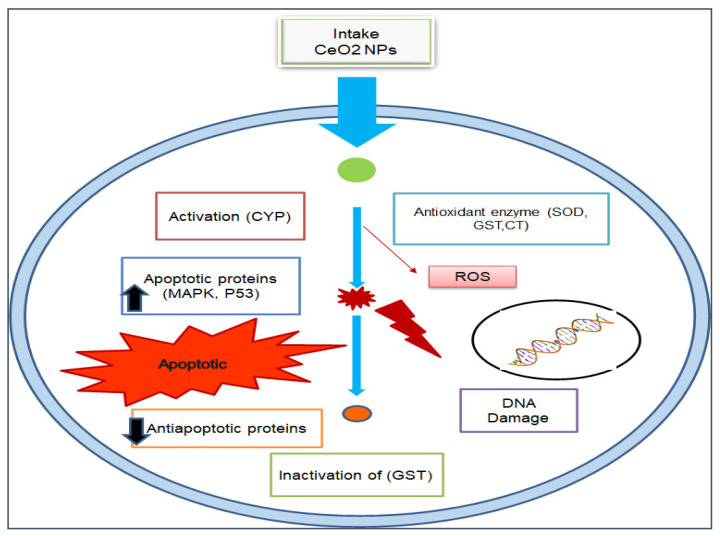
Antioxidant potential of cerium oxide nanoparticles.

Our body constantly produces free radical species through cellular respiration and other processes due to oxidation. The body’s natural antioxidant system continually works to diminish and balance oxidative species numbers inside the body. However, in the case of ailments or any disorder, when the natural antioxidant system fails to compensate for ROS production and to diminish them, an oxidative stress condition arises in the body. In the case of oxidative stress, free radicals or singlet electron-carrying species bind to proteins, mRNA, enzymes, and endomembrane systems and accelerate their lipid peroxidation or nonselective bonding, which changes their chemical nature and properties. The antioxidants interact with free radical species, break their cascade through pairing, and provide protection against oxidative damage (Figure 4). Natural antioxidant systems maintain the balance between the production and degradation of free radical species. Moreover, antioxidants play a significant role in treating oxidative stress, degenerative disorders, and autoimmune disorders such as arthritis (it is a severe joint disease in which body cells themselves destroy mucilaginous material in joints). According to their nature, we categorize antioxidants into two forms: (1) synthetic antioxidants that are taken orally or intramuscularly and (2) natural antioxidants that the body itself produces. However, in the case of surgery, the natural antioxidant system becomes weak, and the content of free radical species increases in the body. However, we have discussed nanotechnology and CeO_2_ NPs and their importance in medicine in the above paragraphs. CeO_2_ NPs are unique because of the free space on their surface. They bind to oxidative species and reduce to Ce^+3^ from Ce^+4^ oxidation states. We have also been discussing the green synthesis of NPs and their medicinal importance. However, it is essential to note that plant-based NPs contain various functional groups on their surfaces that enhance their antioxidant potential, biodegradability in living systems, excellent stability, targeted delivery, and controlled and targeted release of medicinal material [65].

**Figure 5:**
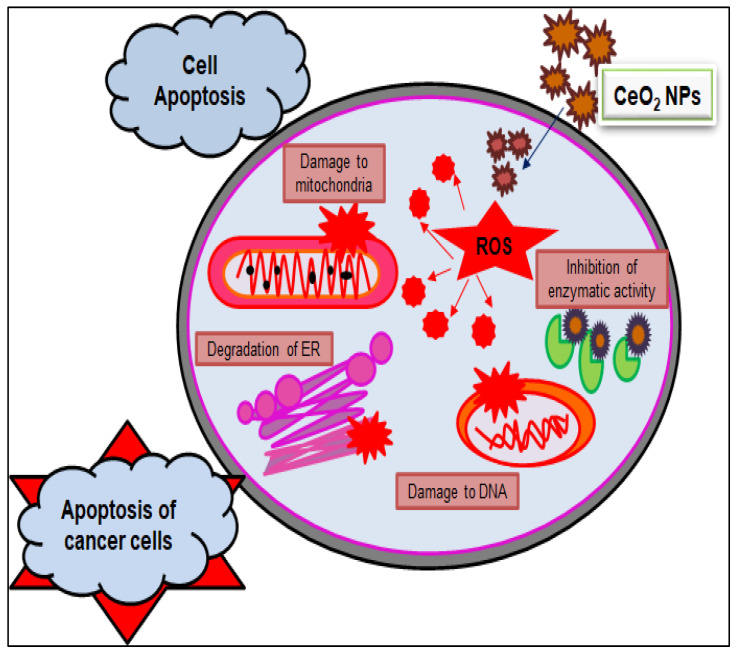
Anticancer mechanistic action of cerium oxide nanoparticles.

GS-CeO_2_ NPs are well-known antioxidants, as documented in previous research [65]. CeO_2_ NPs have excellent antioxidant activity due to their oxidation states varying between Ce^+3^ and Ce^+4^ in an aqueous solution. CeO_2_ NPs exist in Ce^+4^ (oxidized form) in an aqueous solution, but they reduce to the Ce^+3^ form by absorbing superoxide species. Phyto-fabricated CeO_2_ NPs using *Ceratonia silique* L. leaf extract were found to be good scavengers for eliminating ABTS and DPPH superoxide free radicals species in minimum time [3]. Additionally, polyethyleneimine- and glutaraldehyde-loaded nanoceria interact with superoxide dismutase and catalase, increase their antioxidant potential, and protect DNA and proteins from oxidative stress [66]. Moreover, citric acid and EDTA-mediated CeO_2_ NPs show notable antioxidant activity against the deadliest free radicals [67]. It was reported that GS-CeO_2_ NPs that were functionalized with an Fe_2_O_3_ coating enhanced their antioxidant potential and also accelerated the scavenging power of natural antioxidant enzymes glutathione peroxidases and superoxide dismutase, which protect body tissues from oxidative stress (Figure 5) [68]. It was also reported that CeO_2_ NPs show potential wound healing properties in diabetic patients, and recovery time is lessened [69]. Additionally, poly-lactic-*co*-glycolic acid-coated CeO_2_ NPs changed the pH of cancer cells and denatured their enzyme structures [70]. It has been previously discussed that the antioxidant potential of GS-CeO_2_ NPs may be due to functional groups that coat the surface of NPs; this potential comes from plant secondary metabolites [28]. Many researchers have suggested that the use of CeO_2_ NPs in dietary supplements will be effective in balancing antioxidant levels in the body. The biocompatible nature of GS-CeO_2_ NPs makes them important components for food packaging, which currently uses synthetic antioxidants. Phyto-synthesized CeO_2_ NPs have numerous applications in skincare, sunscreen, and skin-whitening products [28].

## 5. Green Cerium Oxide Nanoparticles as Effective Anticancer Agents

Cancer is ranked as the leading cause of death worldwide, as reported by the World Health Organization (WHO) in 2019 [71]. It has been reported that the number of deaths due to cancer worldwide is 19.3 million (18.1 million excluding non-melanoma skin cancer) and 10 million (9.9 million excluding non-melanoma skin cancer) (Sung et al., 2021). There are various forms of cancer: stomach, lungs, cervical, throat, thyroid, prostate, brain, and breast cancer. The most common types of cancer in humans arise due to mutations in proto-oncogenes, which can be radioactively and chemically induced [72]. Some types of cancers are sex-specific; for instance, prostate cancer is most common among men, and breast cancer is most prevalent among women [73]. The Global Cancer Observatory (GCO) website tabulated 36 types of cancer worldwide according to sex and age. The GCO reported that breast cancer cases are high and increasing more than any other cancer globally [71]. Basically two forms of cancer are found: (1) tumors that do not spread, in which abnormal masses of tissues are formed in any one part of the body, and (2) malignant cancer, which spreads vigorously day after day throughout the body [74]. It is difficult to stop the proliferation of cancer cells. Treatments such as surgery, chemotherapeutics, and radioactive therapy are commonly used to kill cancer cells [75]. In addition, hormones and immunotherapy are also utilized to eliminate cancer cells or stop their proliferation. However, these treatments give rise to abnormalities in the patient’s body and, at the same time, also damage normal cells in the patient’s body. These treatments further weaken patients’ immune systems and make them more sensitive to other infections [76]. Cancer cells carry special morphological features that are different from normal cells, such as high blood and lymph vessel proliferation, called angiogenesis and lymph angiogenesis, because uncontrolled spread requires a constant and uninterrupted supply of oxygen and blood [77]. Nanotechnologists utilize the special morphological features of cancer cells to develop targeted, selective, and precisely effective nano-drugs [78]. Nanobiotechnology has a lot of potential to introduce new therapies for the detection and destruction of cancer cells. Targeted drug delivery decreases the chance of drug toxicity. GS CeO_2_ NPs are important tools for site-specific drug delivery [79]. Plants are factories of secondary metabolites such as glycosides, alkaloids, saponins, tannins, flavonoids, polysaccharides, and phenols. Plants use these chemicals for protection against multiple stresses. Nanotechnologists utilize these secondary metabolites as reducing, stabilizing, and capping agents. Organic polymer coatings decrease the toxic potential of CeO_2_ NPs and enhance their medicinal potential [80]. One study revealed that GS-CeO_2_ NPs with an average diameter of 30 nm at 250 µg/mL alleviate ROS levels and cause apoptosis. It was later explained that CeO_2_ NPs activate tumor suppressor P53 protein content [81]. Phyto-fabricated CeO_2_ NPs using the leaf extract of *Bryophyllam daigremontianum* are known to decrease the viability of the MCF 7 breast cancer cell line. It was explained that CeO_2_ NPs denature enzymes and arrest the cell cycle. Another study showed that cancer cells were severely impacted by *Origanum majorana* L. leaf extract-mediated CeO_2_ NPs at 125 µg/mL with an average size between 10–70 nm by the formation of the apoptotic body in MDA-MB-231 cancer cells and induced chromatin condensation, mitotic arrest, and fragmentation [28]. It was also reported that *Justicia adhatoda* L. leaf extract-based CeO_2_ NPs doped with Ag and Au have excellent potential to kill HeLa cancer cells. It was revealed that CeO_2_ NPs at 100 µg/mL significantly arrest cell proliferation in cancer cells by increasing antioxidant activity and limiting the expression of domain-binding proteins [82]. Another study revealed that *Ziziphus jujuba* fruit extract-mediated spherically shaped CeO_2_ NPs at 400 µg/mL with 10 nm diameter penetrated colon cancer cells (HT-29) and induced chromatin condensation, chromosome intermingling, and fragmentation of chromosomes [83]. Similarly, another study observed that biogenic spherically shaped CeO_2_ NPs with 34nm diameter worked like nano-scissors in erythroleukemia cells (WEHI 164). It was revealed that CeO_2_ NPs at 250 µg/mL activate several mitochondrial pathways to induce apoptosis and, finally, autophagy [84].

Furthermore, by exploring the anticancer and cytotoxic potential of CeO_2_ NPs, a new chemotherapeutic nano-drug can be prepared. GS CeO_2_ NPs enter cancer cells through receptor-mediated endocytosis. Malignant cancer cells contain specific pH conditions, and CeO_2_ NPs induce the formation of reactive oxygen species, rupture mitochondrial membranes, and induce the leakage of mitochondrial proteins. On the other side, oxidative stress disrupts the endoplasmic reticulum membranous structure, preventing the binding of ribosomal RNA and halting the translation process. Cellular stress activates many molecular pathways, enhanced EGFR and MAPK/ERK cause cellular apoptosis, and KRT16 reduces cytoskeletal integrity. Nrf2 activates the production of NOO1, HO-1, G6PD, PGD, TALDO1, and TKT, which disturb cell homeostasis via oxidative and inflammatory stress signaling. The activation of these cellular pathways through GS CeO_2_ NPs impairs cell replication machinery, preventing growth-stimulating signaling in the vicinity of cancerous cells (Figure 4). Moreover, GS CeO_2_ NPs reduce cancer cell proliferation and the growth signaling machinery. In the end, disruption of cellular pathways causes the condensation of chromatin, fragmentation of DNA, and denaturation of enzymes and proteins. It was also reported that *Salvadora persica* leaf extract-mediated cerium oxide nanoparticles at 500 µg/mL eradicate (MCF-7) breast cancer cells by binding to the protein using their electrons and halt the further transcription of mRNA [85]. Further, chitosan-loaded cerium oxide nanoparticles were used against 549 and HFF cells, and it was revealed that these nanoparticles induced cell breakage and lipid peroxidation in these cancer cell lines [86]. Figure 5 illustrates the general mechanistic action of cerium oxide nanoparticles as anticancer agent.

## 6. Green Cerium Oxide Nanoparticles as a Potential Drug Delivery Vehicle

Transporting medicinal material to the targeted site in a specific amount is essential to treat disease. The Centers for Disease Control and Prevention (CDC) and National Poison Data System (NPDS) data demonstrated that approximately 10 million deaths occurred worldwide due to drug poisoning, heavy dosages, or nonspecific delivery [87]. Some common pathways utilized for drug administration are enteral (oral administration) and parenteral (muscular or vein injections). The issues that arise during delivery through these pathways are that the drug is equally delivered to both healthy and unhealthy tissues, and a small proportion of drugs are delivered to the targeted site. Drug toxicity, low therapeutic efficiency, and drug resistance are common issues that arise. Drug toxicity due to conventional nonspecific drug administration methods is a major challenge in treating diseases and the overall success ratio [75]. Nanomaterials gained attention when researchers introduced nanomedicine to the market. Nanomaterials are good drug delivery agents due to their site-targeted and amount-specific drug delivery [88]. Nanomaterials can be designed according to desire and requirement [89]. There are many techniques available for nanomaterial preparation, but we prefer the method that is less toxic, less allergenic, easy, safe, and cost-effective, as discussed previously. The green synthesis of nanomaterials using medicinal plants gained the attention of researchers in the last two decades. Currently, various kinds of nanomaterials are synthesized by many pharmaceutical companies. GS CeO_2_ NPs are considered excellent biocompatible agents compared with other nanomaterials, such as Se, Ag, Zn, Cu, and Cd, due to their marvelous biocompatibility and degradability in the living system. Moreover, GS CeO_2_ NPs emerged as novel drug carriers due to their unique photoelectric, catalytic, and optical properties [28]. GS CeO_2_ NPs work as excellent carriers for genes, enzyme inhibitors, biomacromolecules, and bioligands. In some cases, nanomaterials agglomerate with blood cells or lymphocytes, which decreases their medicinal efficiency, bioaccessibility, and bioactivity. However, surface modifications of nanomaterials are remarkable ways to enhance their efficacy during delivery. The coating of CeO_2_ NPs with bioactive ligands enhanced their biocompatibility and synergistically increased their medicinal potential [5]. In a research study, CeO_2_ NPs were coated with Benzyl isothiocyanate (BITC) to deliver the drug to breast cancer cells (MDA MB-231), and it was noted that CeO_2_ NPs wrapped inside BITC successfully reached targeted breast cancer cells [90]. CeO_2_ NPs were synthesized with *Zingiber officinale* aqueous extract functionalized with polyvinyl alcohol hydrogel. It was noted that polyvinyl hydrogel accelerates ROS production in cancer cells. After administration, CeO_2_ NPs and polyvinyl alcohol hydrogel separate, and both synergistically reduce the viability of cancer cells [90].

Furthermore, polyamine-modified CeO_2_ NPs can efficiently deliver Pilocarpine to the ciliary body in the case of glaucoma (ocular disorder) without any systemic inflammation or damage to tissue integrity. There are multiple functions performed by CeO_2_ NPs: firstly, therapeutic against ROS and glaucoma, and secondly, intraocular pilocarpine delivery to ciliary bodies. The hollow surfaces of CeO_2_ NPs were coated with chitosan and ZM241385. The CeO_2_ NPs exhibited effective control in inhibiting ROS, along with delivering Pilocarpine to glaucoma cells and downregulating pro-inflammatory agents such as chemokine (MCP-1) and cytokine (IL-6). CeO_2_ NPs were coated with the organic material chitosan to decrease nonspecific interactions with biological surfaces and with ZM241385, which is a high-affinity adenosine A2A antagonistic receptor that specifically binds to A2AR subtype ciliary body tissues [91]. Moreover, the pretreatment of isolated islets with CeO_2_ NPs can protect them from free radical scavengers and reduce apoptosis until transplantation [92]. Triphenylphosphine-modified CeO_2_ NPs were also reported as a carrier to deliver the drug atorvastatin. They were well-engineered to deliver the drug to acute kidney injury (AKI). Experimental results revealed a considerable reduction in tubular apoptosis, necrosis, and toxicity [93]. Another study revealed that sulfobetaine-loaded CeO_2_ NPs were found to be deadliest against tumors by inducing apoptosis and denaturing membrane proteins [94]. Another example of using bioligands is Carboxybenzenesulfonamide delivery with CeO_2_ NPs, attached via the linker epichlorohydrin, which blocked human carbonic anhydrase (hCAII) activity and induced denaturation of protein structures [94]. Overall, the activities of CeO_2_ NPs still need to be explored. Moreover, due to the biocompatible nature of GS CeO_2_ NPs, it is not difficult to assume that CeO_2_ NPs will be considered a targeted vehicle for drug and gene delivery in the future.

## 7. Antidiabetic Potential of Green Cerium Oxide Nanoparticles

Diabetes is the unnatural buildup of glucose in the body. Diabetes is a chronic metabolic disorder. There are many causes of diabetes, either mutation in genetic material, lack of walking or exercise, long-term depression, unhealthy food, obesity, or islet damage, either genetically or due to long-term allopathic medication. In developed countries where life is critically busy, many people have unhealthy food habits, such as the intake of fast food, alcohol, and soft drinks with high sugar and lack of exercise or walking. These habits have become major issues contributing to diabetes (hyperglycemia). It is reported by the World Health Organization (WHO) that more than 1.5 million deaths globally are due to hyperglycemia, and it is estimated that, in 2030, they will exceed 366 million [95]. The situation has become worse in developing countries, where the basic needs of people’s lives are difficult to fulfill. In these conditions, diabetic patients cannot afford primary care and medicine. According to a WHO survey in 2019, 463 million people (79%) who lived in developing countries had diabetes. It is estimated that at the end of 2045, 700 million people (84%) will be diabetic patients [96]. Diabetes due to genetic mutations is found less often compared with diabetes that is related to an unhealthy lifestyle, eating habits, low physical activity, depression, and genetic complications [97]. However, adopting remedies such as daily checkups of blood glucose levels, blood pressure, and monthly analysis of glycosylation of hemoglobin can be helpful in avoiding complications related to diabetes [95]. There are two types of diabetes: (1) type-1 diabetes is a genetic disorder involving abnormal pancreatic beta cells that are not capable of producing insulin, and in the case of (2) type-2 diabetes, pancreatic islets produce insulin, but body cells do not respond to insulin, which helps to absorb glucose inside the cells, and due to this high level of glucose, it accumulates in the blood [97]. Insulin is a peptide hormone that causes glucose to be transported to and stored in cells. The lower content of insulin and a very high amount of glucose raise blood glucose levels, which damage other body organs. Insulin is key to unlocking cells and absorbing glucose from the blood [98]. Insulin is injected subcutaneously into the patient’s body. The daily administration of insulin therapy over a long period of time accelerates cardiovascular, neuronal, hepatic, and kidney problems [97]. We have previously discussed the antioxidant potential of CeO_2_ NPs. CeO_2_ NPs effectively bind to singlet electron-carrying species. This is why they are considered effective antioxidant agents and can be good antidiabetic agents by protecting pancreatic islets from oxidative damage. According to the Food and Drug Authority (FDA), cerium is a highly biocompatible metal with high antioxidant power. Many pharmaceutical companies are currently working on GS CeO_2_ NPs for their excellent biocompatibility, biodegradability, and non-toxic and antioxidant nature (34). Moreover, plant species that contain antioxidant potential and therapeutic compounds are extensively utilized by pharmaceutical industries. These therapeutic compounds are obtained from plants and are considered highly biocompatible, bioaccessible, and non-toxic to living tissues compared with chemical therapeutic agents (86). For example, due to their reducing power, *Lawsonia intermis* L. leaf extract-mediated CeO_2_ NPs were applied to diabetic rats. Streptozotocin was used to induce diabetes in albino rats. There was a significant reduction in oxidative radical production and triglyceride levels, and the regrowth of pancreatic cells was observed at different doses of NPs [99]. Streptozotocin (STZ) is an antibiotic produced by *Streptomyces achromogenes* bacteria. STZ is used to degenerate pancreatic beta cells and decrease antioxidant SOD, CAT, and GR levels in rats. Similarly, pretreatment of pancreatic β-cells with CeO_2_ NPs before transplantation prevents islets from apoptosis in albino mice [92]. Enzymatic inhibitors’ mimetic activity of nanoceria reduced IL-6 and TNF-α cytokine content and decreased p65-NF-κB expression. Further, *Nrf2* gene expression stimulates pancreatic cells’ antioxidant potential [100]. In the case of diabetic wounds, CeO_2_ NPs were linked with micro-RNA (which is degraded in diabetic patients and causes a chronic wound). These linkers were not injected directly into diabetic mice’s bodies, but a biomaterial was prepared, namely, zwitterion cryogel (the gel was synthesized below freezing temperature). Cryogel was laden with CeO_2_ NPs, and miR146a was applied topically to wounds. The wound was observed continuously for one week, and in that short period, the wound was found to be very well healed [101].

## 8. Green Cerium Oxide Nanoparticles as Effective Potential Antimicrobial Agents

**Figure 6:**
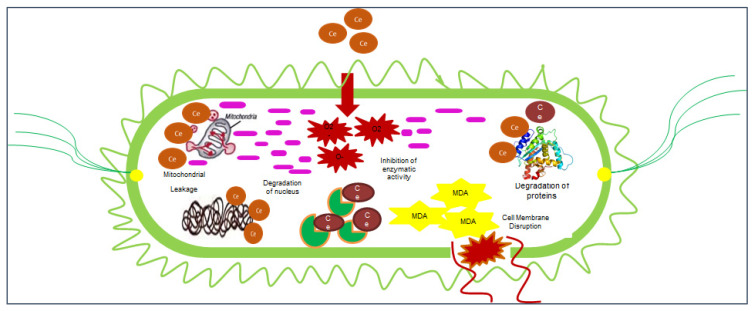
Antibacterial mechanism of action of cerium oxide nanoparticles.

Our environment is surrounded by prokaryotic species. The number of prokaryotes is a thousand times higher than the number of eukaryotes in the world [18]. Human life without prokaryotic species is not possible on earth [102]. They have an impact on our lives, both positively and negatively. Bacteria are unicellular prokaryotic microorganisms. Bacteria are found everywhere on the earth, including in hot springs, permafrost, saline water, and acidic environments [21]. Bacteria also inhabit both the inside and outside the bodies of living organisms. The bacterial flora comprises bacteria in an animal’s body. The bacterial floras of animals’ bodies are soldiers that protect animals from the harmful effects of disease-causing or toxin-releasing microorganisms and control their reproduction rate [103]. However, this normal body flora enters a worse phase when the host immune system is weak due to either infection, surgery, or malnutrition [104]. Many bacterial species are responsible for chronic skin infections and surgical wound infections. Various forms of bacteria are an essential part of planet earth [105]. Many species of bacteria are an important part of the food chain and are critical to the continuity of life on earth [106]. Bacteria are equally important for humans, animals, and plants. Approximately 95% of bacteria are harmless, and the remaining 5% cause diseases in living organisms. However, some species are responsible for causing severe diseases in humans, animals, and plants. Bacteria can be categorized according to their size, shape, nature of the cell wall, feeding mechanisms, feeding habits, habitat, and reproduction methods. Common bacterial species are easily categorized by their cell wall. Bacteria are divided into two categories based on their cell wall, either Gram-positive or Gram-negative. Gram-positive bacteria secrete exotoxin, and Gram-negative bacteria secrete endotoxin. Exotoxin-releasing bacteria are considered less severe than endotoxin-releasing bacteria. Endotoxin is released when antibiotics kill bacteria, releasing the toxin in the victim’s body [102]. There are many antibiotics available in the market, but antibiotic resistance has become a precarious issue globally [105]. Antibiotics kill non-resistant bacterial strains, but those strains containing R (resistant) genes are not affected by antibiotics. All resistant species then divide and reproduce, and antibiotics fail to kill resistant bacteria. According to previous research articles, CeO_2_ NPs have the potential to kill many bacterial species (Figure 6). CeO_2_ NPs perform their action on the bacterial cell wall in three ways. Firstly, if NPs have a size of less than 30 nm, they penetrate the bacterial cell, bind to enzymes and proteins, and denature them. CeO_2_ NPs with a size less than 50 nm produce toxicity in cells and hinder various metabolic processes by changing pH, and NPs with sizes less than 100nm induce redox reactions and perforate bacterial cell walls. *Acalypha indica* L.-mediated CeO_2_ NPs perforate and lyse cells of *Escherichia coli* and *Staphylococcus aureus* [107]. *Calotropis procera* (Aiton) Dryand. GS-CeO_2_ NPs at 40 µg/mL were found to potently penetrate biofilm and cleave bacterial colonies. [30]. Similarly, *Aloe vera* L. gel was used to fabricate CeO_2_ NPs at 100 µg/mL and applied to bacterial strains. Scanning microscopic images showed that NPs bind to bacterial enzymes and stop their reproduction [108]. Another study reported that *Justicia adhatoda* L. leaf extract-based CeO_2_ NPs that were doped with Ag-Au at 100 µg/mL were highly effective in removing biofilms formed on heart valves and pacemakers [82]. Dental biofilm has become a major issue worldwide because of the unavailability of clean water for drinking and contaminated and low-quality food products. *Sida acuta* Burm. f. leaf extract-mediated CeO_2_ NPs at 1000 µg/mL were found to be effective in removing dental plaque by easily entering biofilm, causing toxicity and lysing the biofilm. Biofilm also surrounds various fungal species that obtain nutrition from the biofilm and produce poisonous compounds, which protect the biofilm and bacterial colony from antibiotic attack (Figure 6) [109].

## 9. Green Cerium Oxide Nanoparticles as Potential Antifungal Agents

Fungi are a diverse kingdom and are studied separately because of their unique characteristics, which are different from those of other kingdoms, including Monera, Protista, Plantae, and Animalia. Fungi are an essential saprobe of our earth. Fungi are eukaryotic, multicellular, heterotrophic organisms. Fungi can be microscopic or macroscopic. The body structure of fungi is a long thread-like structure called the hypha, and multiple hyphae join to form mycelia. Fungi can be parasitic, saprophytic, or mutualistic [110]. Two distinguishable features make fungi different from plants. First, all fungal species are unable to synthesize their food because fungal cells lack chloroplasts, so they must take in nutrients from their surroundings. The second major difference is the chitin (N-containing polysaccharide) cell wall; this is why fungi are placed in a separate kingdom. Fungi are divided into six different groups based on their method of reproduction [111]. Various species of fungi form mutualistic relationships with plants, such as mycorrhizal associations. The mycorrhizal association is formed between plant roots and fungi. Some fungal species, such as Glomeromycota, provide phosphorus to plants from the soil, and in return, they receive glucose and nutrients from the plants [104]. Another mutualistic relationship that fungal species form is found in lichens, which is a positive association between fungi and algae. Lichens are pioneers of primary succession [112]. Many fungal species, such as yeast, are an essential part of the baking, wine, sauce, and cheese-making industries. Many species of fungi are edible and are used to make curries, such as species of Basidiomycota (mushroom) and Ascomycota (truffle) [113]. Despite their beneficial role, many species of fungi are responsible for many diseases in humans, animals, and plants. There are four notorious species of fungi: Rust, Smut, *Aspergillus flavus*, and *Penicillium*, which severely damage crops and are responsible for huge losses every year. Many species of fungi, including *Aspergillus*, *Penicillium*, *Pneumocystis*, and *Candida albicans*, are major causes of tonsil, esophageal, nasal, mouth, and pulmonary infections [114]. *Olea europaea* L. leaf extract-mediated CeO_2_ NPs at 200 µg/mL were utilized against *Candida albicans* hyphae. It was observed that GS CeO_2_ NPs accelerate lipid peroxidation, perforation, and the leakage of cell material [115]. Another study explained the role of CeO_2_ NPs in eradicating dental plaque in different areas of the mouth. The CeO_2_ NPs eradicate fungal mycelia, which provides a protective covering to biofilm, by penetrating biofilm [116]. Similarly, xanthan-gum-fabricated CeO_2_ NPs were doped with iron salt, and hybrid CeO_2_ NPs were applied against the mycelia of *Candida albicans* and *Fusarium oxysporum*. CeO_2_ NPs at 500 µg/mL blocked cell division in mycelia and condensation of chromatin [117]. *Hyphaene thebaica* (L.) Mart. fruit extract-mediated CeO_2_ NPs were applied against the following fungi: *Fusarium solani*, *Aspergillus fumigatus*, *Aspergillus flavus*, and *Aspergillus niger*. CeO_2_ NPs at 2 mg/mL concentration were found to be effective in inhibiting enzyme catalytic sites and caused the denaturation of enzymes, resulting in the arrest of translation, protein assemblage, and protein folding and in the induction of chitin oxidation [118].

## 10. Cytotoxicity of Cerium Oxide Nanoparticles

The toxicity of NPs is due to the charge on their surface. High-positive-charge-carrying species develop good electrostatic interactions with biological materials. CeO_2_ NPs are popular due to their dual oxidation state, Ce^+3^ to Ce^+4^, which enhances CeO_2_ NPs’ catalytic activity in biosystems [119]. The interaction between GS-CeO_2_ NPs and biosystems is completely based on the surface chemistry of NPs. The charge of NPs basically determines the cellular interaction of CeONPs with organelles and ultimately dictates biological responses [120]. Their nanometer size boosts the penetration power of CeO_2_ NPs into cells and allows them to easily cross the nuclear membrane. This nanosize increases the cell penetration capabilities of CeO_2_ NPs and makes them a powerful tool for antifungal, antimicrobial, pesticide, insecticide, and anticancer activities. The different sizes of NPs play different roles inside the cell. Upon entrance into the cell, radical-hungry species (CeO_2_ NPs) can easily interact with proteins, lipids, and enzymes and also induce the fragmentation of genetic material and lipid peroxidation of the cell membrane and ultimately halt cell metabolic processes [121]. CeO_2_ NPs can enter the cell either through phagocytosis, macro-pinocytosis, or receptor-dependent endocytosis [122]. CeO_2_ NPs contain oxygen vacancies on their surface that show high affinity to electron-carrying species, which is why CeO_2_ NPs accelerate cell membrane damage and the leakage of cell material. One study illustrated that CeO_2_ NPs accelerate the transcription of cytochrome c, which accelerates caspase-3 and caspase-4, which induce apoptosis in cancer cells by targeting mitochondrial proteins, which indirectly reduces the ATP level for cancer cell multiplication [123].

## 11. Conclusions and Future Perspectives

This review explains the immense significance of GS-CeO_2_ NPs in various fields of nanomedicine. Many experimental results demonstrate that CeO_2_ NPs have a high potential to treat chronic disorders. It has been reported that GS-CeO_2_ NPs are nonallergenic, non-toxic, bioaccessible, biocompatible, and biodegradable. CeO_2_ NPs possess an electropositive charge on the surface and vacant spaces and bind free radicals from the environment, so they are considered good antioxidant agents and have the potential to replace many chemically prepared antioxidants in the pharmaceutical industry. Nanotechnology is capable of resolving another issue, antibiotic resistance, which has caused panic for the pharmaceutical industry. Bacteria evolve rapidly and develop resistance to antibiotics. Thus, the discovery of new antibiotics is necessary for coping with bacterial diseases. However, nanomaterials are an essential breakthrough for pharmaceutical industries to deliver the drug more specifically to the targeted point. In the recent past, we have experienced a global viral (COVID-19) pandemic that has threatened human health and civilization, so cerium oxide nanoparticles can also be used for the preparation and commercialization of antimicrobial personal protective equipment because of their outstanding potential antimicrobial efficacy. There is also a need to explore the toxicological and cytotoxic impacts of cerium oxide nanoparticles. Additionally, cerium oxide nanoparticles could have potential applications in agriculture and environmental sustainability; however, there is still a need to explore the application potential and mechanistic actions of cerium oxide nanoparticles.

## Figures and Tables

**Table 1:** Cerium oxide nanoparticle synthesis by using various routes and their biomedical applications.

No.	Plant Name	Plant Part Used	Size of NPs	Activity	References
1	*Calotropis procera*	Flower	20 nm	Biogenic CeO_2_ NPs exhibited important antibacterial activity against *E. coli* and *Pseudomonas*.	[30]
2	*Solanum nigrum* L.	Leaves	20 nm	Biosynthesized CeO_2_ NPs exhibited the highest antibacterial activity against Gram-positive *Bacillus subtilis* and Gram-negative against *E. coli*.	[31]
3	*Aloe barbadensis*	Gel	10 nm	Green CeO_2_ NPs showed high antioxidant potential.	[32]
4	*Olea europaea* L.	Leaves extract	24 nm	Successful inhibition of fungal and bacterial strains.	[33]
5	*Azadirachta indica*	Leaves	50 nm	CeO_2_ NPs exhibited a good photo-degradation rate.	[34]
6	*Gloriosa superba* L.	Leaves	5 nm	CeO_2_ NPs exhibited good photoluminescence and antibacterial activities against Gram-positive and Gram-negative species.	[35]
7	*Citrullus lanatus*	Juice	11.6 nm	Biosynthesized CeO_2_ NPs exhibited good photocatalytic activity and antibacterial potential by causing leakage of the bacterial membrane.	[36]
8	*Prosopis fracta*	Fruit	15 nm	Green synthesized CeO_2_ NPs showed cellular toxicity against colon cancer cells.	[37]
9	*Prosopis fracta*	Aerial parts (leaves, branches)	30 nm	Biosynthesized CeO_2_ NPs were found to be less effective against HT-29 cancer cells.	[38]
10	*Camellia sinensis* L.	Leaves	5 nm	Biogenic CeO_2_ NPs were found to be protective against the oxidation of hepatic inflammation and oxidation of hepatic cells.	[38]
11	*Humicola* sp.	*Fungus mycelia*	5 nm	Biosynthesized CeO_2_ NPs were found to be highly stable and did not agglomerate in an aqueous solution.	[39]
12	*Salvadora persica* L.	Whole plant extract	20 nm	Green synthesized CeO_2_ NPs were found to be effective against a breast cancer cell line (MCF-7).	[119]
13	*Musa sapientum* L.	Fruit	13 nm	Biosynthesized CeO_2_ NPs were found to be good sun-protective agents and anticancer agents against a lung (A549) cancer cell line.	[40]
14	*Acalypha indica* L.	Leaves	15–30 nm	Biogenic CeO_2_ NPs showed antibacterial behavior against Gram-positive and Gram-negative species.	[41]
15	*Brassica napus* L.	Pollen grains	4 nm	Green CeO_2_ NPs destroyed ovarian cancer cells (A2780).	[42]
16	*Aspergillus niger*	*Fungus mycelia*	5–20 nm	Green CeO_2_ NPs exhibited high insecticidal potential against *Aedes aegypti* and antibacterial activity against *Streptococcus pneumonia*, *Bacillus subtilis.*	[43]
17	*Origanum majorana* L.	Leaves	70 nm	Green synthesized CeO_2_ NPs could express SOD, CAT, POX, and antioxidant activities and were found to be highly cytotoxic against the MDA-MB-231 cancer cell line.	[28]
18	*Prosopis juliflora*	Leaves	3.7 nm	Green synthesized CeO_2_ NPs were highly effective in killing both Gram-positive bacteria (*Staphylococcus aureus*, *Streptococcus pneumonia*) and Gram-negative bacteria (*Pseudomonas* *aeruginosa*, *Proteus vulgaris*).	[44]
19	*Aloe vera* (L.)	Leaves	2–3 nm	Biogenic CeO_2_ NPs were found to be highly antioxidant agents.	[45]
20	*Petroselinum crispum*	Fruit	25 nm	Green CeO_2_ NPs exhibited high antioxidative activity against various stresses in agricultural plants.	[46]
21	*Musa sapientum* L.	Peel extract	4–13 nm	Green CeO_2_ NPs exhibited high photocatalytic activity.	[47]
22	*Acorus calamus* L.	Rhizome extract	22.03 nm	Biogenic CeO_2_ NPs showed good antibacterial activity against Gram-positive and Gram-negative species.	[48]
23	*Moringa oleifera*	Seed	30 nm	Green CeO_2_ NPs were found to express suitable insecticidal activity.	[49]
24	*Hibiscus Sabdariffa* L.	Flower	3.9 nm	Green synthesized CeO_2_ NPs were found to be highly effective chelating agents.	[50]
25	*Amomum subulatum*	Seeds	0.5 µm	Green CeO_2_ NPs were found to be highly effective against MRSA, methicillin-resistant *S. aureus* infection, which primarily affects animal mammary glands.	[51]
26	*Aloe vera* (L.)	Leaves	7–12 nm	Green CeO_2_ NPs showed good optical properties at different concentrations of nanoparticles.	[52]
27	*Sida acuta*	Leaves	8.2 nm	Green synthesized CeO_2_ NPs disrupted the cell membrane of *E. coli* and killed bacteria.	[26]
28	*Rheum turkestanicum*	Whole plant	30 nm	Green synthesized CeO_2_ NPs exhibited photocatalytic and cytotoxic activities against PC12 cell lines.	[53]
29	*Saccostrea cucullata*	Whole mollusk	15 nm	Biogenic CeO_2_ NPs exhibited suitable photocatalytic and cytotoxic activities.	[54]
30	*Ceratoniasilique* L.	Leaves	100 nm	Green synthesized CeO_2_ NPs were found to be effective against the hepatic cancer cell line.	[3]

## Data Availability

Not applicable.
